# Real‐Time Monitoring of Biometric Responses During a 200‐km Ultra‐Endurance Race Across the Desert

**DOI:** 10.1002/ejsc.70026

**Published:** 2025-08-11

**Authors:** Chris J. Esh, Yannis Pitsiladis, Sebastien Racinais, Lee Taylor, Valentin Dablainville, Toaufik Belfekih, Fawzi Bendimerad, Asimina Pitsiladis, Panagiotis Verdoukas, Mark Willems, Nelda Nader, Feriel Dalansi, Paul Grandjean, Maha Al‐Mulla, Nada Aldous, Joseph Dossou, Youmna Elsayed Hassanein, Nada Khater, Herald Miranda, Marco Cardinale

**Affiliations:** ^1^ Aspetar Orthopaedic and Sports Medicine Hospital Doha Qatar; ^2^ School of Sport, Exercise and Health Sciences National Centre for Sport and Exercise Medicine (NCSEM) Loughborough University Loughborough UK; ^3^ Department of Sport, Physical Education and Health Hong Kong Baptist University Kowloon Hong Kong; ^4^ Human Telemetrics Ltd London UK; ^5^ CREPS Montpellier—Font Romeu Environmental Stress Unit Montpellier France; ^6^ DMEM Univ Montpellier INRAE Montpellier France; ^7^ School of Sport, Exercise and Rehabilitation Faculty of Health University of Technology Sydney (UTS) Sydney Australia; ^8^ Human Performance Research Centre University of Technology Sydney (UTS) Sydney Australia; ^9^ College of Health and Life Sciences Hamad Bin Khalifa University Doha Qatar; ^10^ Department of Targeted Interventions University College London London UK; ^11^ Department of Sport, Exercise and Rehabilitation Northumbria University Newcastle Upon Tyne UK

**Keywords:** hyperthermia, hypothermia, real‐time athlete monitoring, thermal physiology, ultra‐endurance

## Abstract

Ultra‐endurance sports challenge athlete health, with these risks exacerbated by environmental extremes and/or remoteness of competition. Therefore, this study aimed to use real‐time monitoring technology to characterise and monitor physiological/biomechanical responses during SAMLA 2023, a 200‐km multidiscipline (swim, run, bike, and kayak) ultra‐endurance race, encompassing cool and warm desert environmental conditions (16°C–28°C). Within a cross‐sectional observational study design, 18 males (total entrants: 318) were instrumented with wearable/ingestible sensors measuring physiological [heart rate and core (Tc)/skin (Tsk) temperature], biomechanical [gait] and location‐based [Global Positioning System (GPS)] metrics. Sensors connected to a smartphone application via Bluetooth, which saved and transmitted data to a cloud‐based dashboard in real‐time. Participants were on‐course for an accumulated ∼668 h. ∼662 h of GPS data were displayed in real‐time with the longest individual data capture of ∼57 h. Physiological/biomechanical data were acquired for x̄: ∼42% (range: ∼38%–∼49%) of the participant on‐course time. Hypo/hyperthermic Tc's were seen (x̄: 37.8°C range: 35.7°C–39.2°C). Tsk (28°C: 11.7°C–38.4°C) in response to the varied in‐race environmental challenges (16°C–28°C ambient temperature) and heart rate (111 b·min^−1^: 37 b·min^−1^–179 b·min^−1^) varied markedly. One participant was hospitalised without presentation in physiological data. Biomechanical data had significant data loss and quality issues and are not presented. Developments in real‐time monitoring technology, acknowledging limitations observed here (physiological/biomechanical data acquisition), may allow combined in‐race GPS and physiological data (e.g., Tc/Tsk) to be used during ultra‐endurance sport to prospectively protect athlete health. GPS/physiological data alone may not identify medical emergencies, and medical teams must remain alert to medical events.

## Highlights

- Athletes can experience significant physiological and thermoregulatory strain in ultra endurance events, even when exercise intensity is relatively low and environmental conditions seem favourable for a safe race from a thermoregulatory stress (heat and cold) perspective.
- The use of real‐time monitoring technology was largely successful providing evidence that in‐race physiological data visualisation is possible across long duration races in remote locations. However, in its current form, it suffers limitations that restrict implementation on a wider scale.
- On‐course medical emergencies will not always present with physiological indicators becoming progressively alarming. Medical teams must remain prepared to respond to medical events even when athletes' biometric data are available in real‐time and do not provide alerts.

## 1 Introduction

Participation in ultra‐endurance events has increased worldwide from < 50,000 ultramarathon runners to > 300,000 over the last 2 decades (Scheer 2019). Multiday ultra‐endurance events cover vast distances across variable terrain, often within remote locations at sea‐level/altitude and across day/night where participants can experience changeable extremes of weather (i.e., heat, humidity and cold) within a race. For example, 21 runners died in 2021 from hypothermia (a further eight were hospitalised) during a 100‐km ultramarathon in China when mild race start environmental temperatures fell rapidly alongside sudden high and cold winds, rain and hail (Hoffman 2021). In contrast, global temperatures are increasing and sporting events are increasingly likely to take place in extreme heat (Esh et al. 2024). In extreme heat, physical activity increases core (Tc), skin (Tsk) and muscle temperature that can result in exertional heat illness (EHI) and/or stroke [EHS (Bouscaren et al. 2019; Esh et al. 2024)].

Clearly, ensuring participant safety and providing appropriate medical services are a necessary; yet, logistically challenging priority within ultra‐endurance sport (Hoffman et al. 2020, 2014), especially, when unexpected rapid changes in environmental conditions occur as seen during the race in China (Hoffman 2021). Using the event in China as an example, the availability of real‐time localised environmental (e.g., temperature/humidity) and participant physiological (e.g., Tc/Tsk temperature) data to race directors and medical teams could have improved response time and decision‐making in this ultra‐endurance event. Real‐time data in ultra‐endurance events, where injury/illness are common (Hoffman et al. 2014), could enable triage and dispatch of medical resource to the precise location of those that need it most. Important, given the variety of health‐related challenges that can present, from nonlife‐threatening blisters and musculoskeletal injuries to serious adverse events, such as exercise associated collapse [e.g., cardiac arrest and EHS], which can be fatal if not treated rapidly (Hoffman 2021; Hoffman et al. 2014).

Advancements in Global Positioning System (GPS) technology have improved athlete tracking and subsequently, the search and rescue capabilities of medical support services during ultra‐endurance events (Hoffman et al. 2018). For example, during a 332‐km ultramarathon through the Cascade Mountains in Washington State, a participant became lost and disoriented due to exhaustion and wandered ∼10‐km off course, the race medical team were able to locate and return the individual safely to the support tents for treatment and safe release (Hoffman et al. 2018). However, in this context, GPS only relays basic location and time‐motion data. Innovative developments in wearable technology now allow for physiological [e.g., heart rate (HR)] and biomechanical (e.g., gait analysis) measurements in‐competition to be recorded and broadcasted in real‐time (Guppy et al. 2023; Muniz‐Pardos et al. 2021). Until recently, such data have only been available retrospectively (i.e., postrace data download/processing) and has generally been limited to Tc and Tsk in‐competition (Singh et al. 2023). Real‐time athlete monitoring could increase the safety of ultra‐endurance events allowing medical teams to assess athlete health (and location) data remotely (Muniz‐Pardos et al. 2021), respond rapidly and triage resource allocation.

A real‐time monitoring ecosystem utilising wearable/ingestible technology has successfully assessed athlete's responses to competition (Guppy et al. 2023) albeit during short duration events (under 3 h). Therefore, it was the aim of this project to explore the feasibility of an experimental real‐time monitoring and visualisation system within the context of the SAMLA ultra‐endurance race in Qatar, a 200‐km multidiscipline (swim, run, bike, and kayak) event where data acquisition and broadcasting were required for ∼57 h (i.e., race cutoff time). Additionally, using this experimental real‐time application, characterise and monitor the physiological and biomechanical responses to the SAMLA ultra‐endurance event in desert cool to warm environmental conditions (16°C–28°C). Given the unique challenges to human physiology, and the diverse physical fitness levels of the participants, completion times typically range from 15–60 h across the range of environments and temperatures recoded in previous SAMLA races.

## 2 Materials And Methods

### 2.1 Participants and Study Design

**FIGURE 1:**
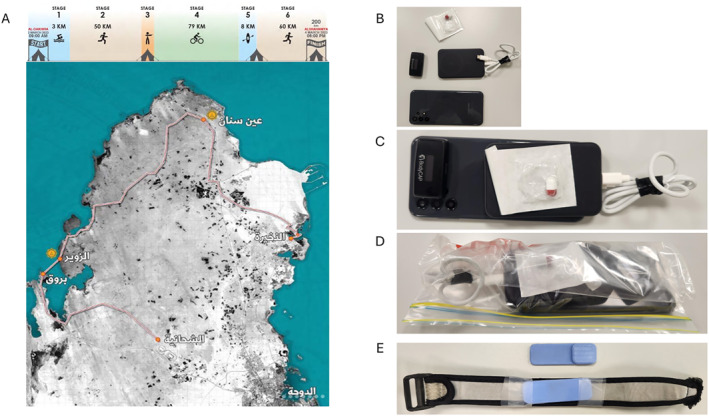
Race map and visualisation of items participants were asked to carry in their race rucksack. Consisting of smartphone with mobile application, powerbank, gateway device and spare core temperature pill.

A cross‐sectional observational and descriptive study design was implemented. Out of 318 entrants, 19 males participating in the 2023 SAMLA ultra‐endurance race were recruited for this project. SAMLA takes place across desert terrain in Qatar (Figure 1A) and is open to male Qatari citizens only. One participant withdrew from the study prior to the start of the race (did not withdraw from the race). The data presented are from 18 individuals [mean (range) age: 35.1 *y* (18 *y*ears–52 *y*ears) and body mass: 80.5 kg (65.8–108.3 kg)]. Procedures were approved by the Aspire Zone Foundation Institutional Review Board (E202301049). Informed consent was obtained from all study participants prior to the start of the race.

### 2.2 Procedures

Participants carried a smartphone (Samsung A13, Samsung, South Korea), powerbank (Anker 21, Washington, USA) and BodyCAP Gateway (BodyCap, Hérouville Saint‐Clair, France), weighing ∼650g, to collect and broadcast data from the wearable sensors (see Figure 1B–D). Powerbanks were changed when necessary. Participants were able to select which of the available sensors to be instrumented with. No participants were excluded from the study if they opted not to be instrumented with one of the sensors.

#### 2.2.1 GPS and Real‐Time Monitoring

All sensors worn throughout the race were connected to a bespoke smartphone application (Sub2, Human Telemetrics, London, UK—not commercially available) via Bluetooth. The smartphone location capabilities were used to provide GPS data that were collated and transmitted by the application. The application transmitted the data wirelessly via the cloud to the real‐time dashboard (Human Telemetrics, London, UK). These processes [outlined previously (Muniz‐Pardos et al. 2021)] were originally designed for use on a smartwatch and were adapted for use on a smartphone to facilitate battery requirements. Cellular connectivity was achieved using SIM cards from Ooredoo, Qatar. Ooredoo, Qatar has full Global System of Mobile Communications (GSM) network coverage across Qatar and a 3G/4G connection was used throughout.

#### 2.2.2 Environmental Conditions

Environmental conditions were measured in real‐time via an application programming interface connection [described previously (Guppy et al. 2023), www.extrema‐global.com, ARTi Analytics BV; Rotterdam, Netherlands]. Due to a server error, the data from this application were not available retrospectively (i.e., data could not be downloaded from the real‐time dashboard). Thus, historical data (timeanddate.com) were used to describe the environmental conditions. Environmental temperature range: 16°C–28°C.

#### 2.2.3 Core and Skin Temperature

Tc was assessed via a telemetric pill and Tsk via an e‐Flex device (Bodycap, Hérouville Saint‐Clair, France). Data from these sensors (radio frequency) are read by a gateway device that translates this into a Bluetooth frequency, which is sent to and read by the mobile application. Only three sensors can be connected to a gateway device; thus, only two pills were activated per participant (the remaining communication channel was used for collecting data from the Tsk sensor). The Tc pill was ingested after race registration (∼1–2 h prior to race start), a four‐to‐five‐hour prerace ingestion time is typically preferred to ensure data reliability [e.g., no impact of water consumption (Bongers et al. 2015)]. However, with only two available pills and participants potentially in the race for 57 h, the decision was taken to prolong in‐race data capture time and avoid early excretion of the pill. The e‐flex was placed under a provided wrist strap (Figure 1E). A second Tc pill was placed in the participants race backpack and was taken if the first pill was excreted during the race. One participant opted to not ingest the Tc pill.

#### 2.2.4 Heart Rate

HR was assessed using a HR monitor and chest strap (H9, Polar, Kempele, Finland), 13 participants chose to wear the HR monitor.

#### 2.2.5 Gait Analysis

A foot‐worn inertial sensor (ORPHE Inc., Tokyo, Japan) was attached to the participants shoelaces during the two run phases only, 14 participants wore sensors on both shoes and four participants on the left foot only. Sensors were removed at the end of Run 1, charged and then put back onto the participants shoe(s) at the start of Run 2. The ORPHE sensors were used to measure cadence, flight time and stride length. The sensors were impacted by the desert sand/dust and participants regularly dousing themselves with water. Following initial analysis, these data are not presented due to significant data loss and poor data quality.

### 2.3 Statistical Analysis

Data were manually assessed for artifacts (e.g., Tc pill data affected by water consumption) and any nonsensical or biologically impossible data were removed (e.g., a Tc change ≥ 0.3°C in ≤ 30 s and consistent HR value for ≥ 5 s) using Microsoft Excel (Microsoft Corps, Redmond, WA, USA). If two Tc pills were recording data from a participants simultaneously, data from the first pill ingested was used. No inferential statistical comparisons have been made given: (1) the observational nature of this study; (2) participants were completing the different disciplines within the race across the variable environmental conditions during the race and (3) stage length differences and participant numbers within each stage varied as participants withdrew from the race and; (4) availability of data retrospectively varied between participants (see *n* presented in Table 1). Data are only presented descriptively as mean and range (min–max). Time spent running (movement speed ≥ 7 km/h), walking (movement speed from 0.5 km/h to 6.9 km/h) and not moving (movement speed ≤ 0.49 km/h) were assessed during the run stages.

**TABLE 1:** Retrospective data availability for sensors used.

	Total data capture time (hhh:mm:ss)	Retrospective data availability (hhh:mm:ss)	Retrospective data availability (% on‐course time)	Longest single data capture (hh:mm:ss)
Tc	610:57:08	297:16:00	49	41:49:00
Tsk	668:29:12	255:33:00	38	41:49:00
Gait	365:09:21	142:15:09	39	N/A
HR	337:22:08	119:33:55	35	N/A

## 3 Results

Mean (range) data across the whole race are presented within the text below. Within stage mean (range), data are presented in Tables and Figures.

### 3.1 Real‐Time Monitoring

**FIGURE 2:**
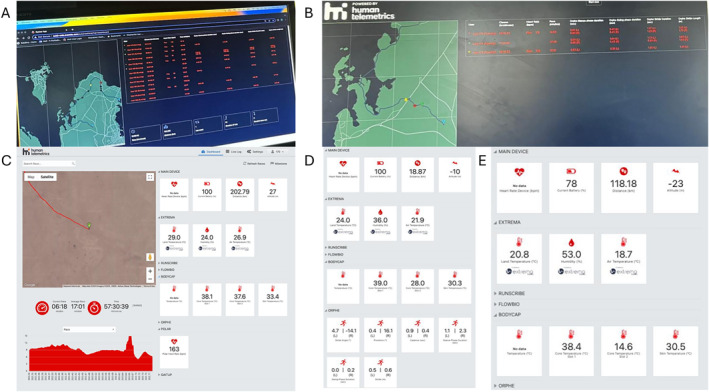
Data visualisation from the live dashboard. (A–B): multiple user dashboard showing data from all participants on the course in real‐time. (C–E): individual user dashboard from points across the race in real‐time. NB: these visualisations are photographs and/or screenshots of the live dashboard taken during the SAMLA race. They are only presented to illustrate how the real‐time data were visualised and do not present data of significance to the results of this study.

Other than approximately six hours of data that were unavailable due to battery power for one participant, GPS data were visualised in real‐time throughout the duration of the event for all participants (Figure 2 depicts visualisations of the live dashboard), a combined total of 668:29:12 (hhh:mm:ss) of data were captured. The longest individual data capture was 57:32:04 (*n* = 6 exceeded 48 h).

It was not possible to determine with accuracy the amount of physiological and biomechanical data that were displayed in real‐time. Technological issues [including but not limited to sensor connectivity issues, participants excreting (Tc pill),losing (Tsk sensor) andnot wearing (HR) sensors] prevented physiological/biomechanical data being displayed in real‐time for the duration of the race. Additionally, if a sensor lost connection with the Sub2 application, the live dashboard continued to display the data from the last point of connection. Table 1 provides details of the data that were available for analysis following data cleaning, providing an estimate of the time data were displayed in real‐time. One participant's data were visualised in real‐time but could not be downloaded from the real‐time dashboard due to a server error.

### 3.2 Core Temperature

Mean race start Tc was 37.3°C (36.5°C–37.9°C) and in‐race was 37.8°C [minimum mean: 37.4°C and maximum mean: 38.1°C (range: 35.7°C–39.2°C)]. Tc during daytime and nighttime hours were similar [Day: 37.8°C (35.7°C–39.2°C), Night: 37.8°C (36.3°C–39.1°C)]. The below demonstrates the percentage of time Tc data that were observed to be within the outlined ranges:

1. ≤ 35.9°C (*n* = 1): 0.05%
2. 36.0°C–36.9°C (*n* = 12): 8.0%
3. 37.0°C–37.9°C (*n* = 13): 46.4%
4. 38.0°C–38.9°C (*n* = 13): 45.0%
5. ≥ 39.0°C (*n* = 4): 0.6%

Health and/or performance detriments can occur when time is spent with a Tc ≤ 35.9°C (M. J. Tipton et al. 2017), ≥ 38.5 or ≥ 39°C (Girard et al. 2015; Nybo et al. 2014; Roberts et al. 2023). The below demonstrates the individual time Tc data that were observed to be above/below the outlined Tc:

1. ≤ 35.9°C (*n* = 1): 0.8%
2. ≥ 38.5 (*n* = 9): 13% (1%–24%)
3. ≥ 39°C (*n* = 4): 1.5% (0.5%–2.5%)

### 3.3 Skin Temperature

Mean start Tsk was 31.7°C (26°C–35.4°C) and across the whole race was 28°C [minimum mean: 20.6°C and maximum mean: 30.4°C (range: 11.7°C–38.4°C)].

### 3.4 Core to Skin Temperature Gradient

The mean Tc‐to‐Tsk gradient at race start was 5.7°C (3.6°C–10.1°C) and across the whole race was 9.7°C [minimum mean: 7.6°C and maximum mean: 11.6°C (range: 1.6°C–19.6°C)].

**FIGURE 3:**
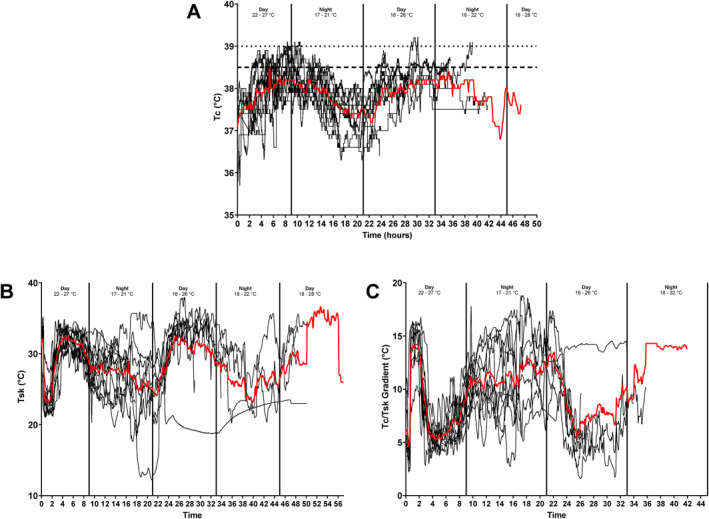
Core and skin temperature responses and core to skin gradient across the race. Black lines are data from each individual participant. Thicker red line is the mean. (A): core temperature, (B): skin temperature and (C): core‐to‐skin temperature gradient.

All Tc, Tsk and Tc‐to‐Tsk gradient data are presented in Table 2 and Figure 3.

**TABLE 2:** Tc and Tsk responses to each stage.

	Stage 1: 3‐km swim	Stage 2: 50‐km run 1	Stage 3: 79‐km bike	Stage 4: 8‐km kayak	Stage 5: 60‐km run 2
Tc (°C)	*(n = 13)*	*(n = 13)*	*(n = 10)*	*(n = 8)*	*(n = 8)*
Start	37.4 36.0–38.5	37.2 36.0–37.9	37.6 37.0–38.3	37.5 37.0–38.2	37.5 36.6–38.5
Mean	37.3 35.7–38.6	38.0 36.0–39.1	37.8 36.3–38.9	37.5 36.0–38.2	37.9 36.5–39.2
Peak	37.7 36.9–38.6	38.5 36.0–39.1	38.4 37.2–39.0	37.9 37.4–38.2	38.7 37.8–39.2
Change	0.6 0.0–2.6	1.5 0.4–2.5	0.9 0.4–1.3	0.4 0.0–0.9	1.1 0.0–1.8
Tsk (°C)	*(n = 12)*	*(n = 12)*	*(n = 8)*	*(n = 7)*	*(n = 4)*
Start	32.1 28.2–33.8	29.6 23.9–34.3	29.4 25.6–32.7	29.1 27.3–33.6	27.7 21.6–31.5
Mean	30.8 20.1–36.9	29.1 19.2–35.1	28.9 17.2–38.4	27.8 22.2–33.6	30.8 20.1–36.9
Peak	33.3 28.5–35.3	34.1 33.0–35.1	34.4 31.1–38.4	30.3 27.8–33.6	35.2 33.9–36.9
Change	1.3 0.0–4.7	4.3 0.4–11.0	5.0 0.1–11.7	1.2 0.0–4.1	7.5 4.3–15.3
Tc/Tsk gradient (°C)	*(n = 10)*	*(n = 10)*	*(n = 7)*	*(n = 3)*	*(n = 2)*
Start	5.2 3.5–9.2	7.5 3.1–13.9	7.7 5.2–11.8	8.7 7.3–9.8	7.4 7.0–7.7
Mean	11.6 1.6–16.5	6.2 2.7–13.9	9.8 4.2–15.8	10.0 7.1–11.7	10.9 8.5–13.3
Peak	14.7 10.7–16.5	8.9 6.5–13.9	11.9 9.1–15.8	11.0 8.2–12.8	13.5 10.7–16.3
Change	9.5 6.7–11.9	1.4 0.0–5.5	4.1 2.5–6.3	2.4 0.8–3.3	6.2 3.0–9.3

*Note:* Values are expressed as mean and range (*n* = number of participants for each measure per stage). The ‘Change’ in Tc is calculated as Stage Peak Tc–Stage Start Tc.

Abbreviations: Tc (core temperature) and Tsk (skin temperature).

### 3.5 Heart Rate

HR sensor connectivity was intermittent; due to discomfort, some participants removed the chest strap and/or moved it away from the chest during the race. From the available data (*n* = 9 participants), mean start HR was 102 b·min^−1^ (89 b·min^−1^–113 b·min^−1^) and during the whole race was 115 b·min^−1^ [minimum mean: 104 b·min^−1^ and maximum mean: 136 b·min^−1^ (range: 37 b·min^−1^–179 b·min^−1^)]. Mean percentage of estimated HRmax (calculated as: 220–age) was 63% (53%–75%).

### 3.6 Health and Performance

Of 318 starters, 136 (43%) did not finish (DNF). The winning race time was 17:35:58 (see Table 3 for mean finishing times and race speeds). Of the current study participants, one participant finished inside the top 30. Nine study participants DNF: three failed to reach the finish before the race cutoff time (57 h), five voluntarily withdrew from the race and one required hospital treatment for exhaustion following an on‐course collapse (discharged within 24 h). Time spent walking, running and not moving during the run stages are presented in Table 3.

**TABLE 3:** Performance based data.

Finishing time (all race data)	
All race finishers	40:48:39 (17:35:58–57:00:55)
Top 3 finishers	17:52:52 (17:35:58–18:16:09)
Study participant finishers	41:32:43
Mean speed (km/h)	
All race finishers	5.4 (3.5–11.3)
Top 3 finishers	11.1 (10.9–11.3)
Stage (study participants)	Mean speed (km/h)
Swim	2.0 (1.5–2.4)
Run 1	5.0 (3.9–7.5)
Bike	10.4 (7.6–13.5)
Kayak	4.1 (3.2–5.2)
Run 2	4.6 (3.6–5.9)
Run stage characteristics (study participants)	
Run 1	% of time
Walk	72.4
Run	17.3
Not moving	1.4
Run 2	
Walk	79
Run	6.3
Not moving	1.2

*Note:* Values are expressed as mean (range). Range not provided for study participant finish time and mean speed to avoid participant identification. Percentages for run stage characteristics do not add up to 100% due to not all data for each stage being available retrospectively. 91.1% of the total time spent on‐course was available for Run 1% and 86.5% for Run 2. Walking = movement speed from 0.5 km/h to 6.9 km/h, running movement speed ≥ 7 km/h and not moving = movement speed ≤ 0.49 km/h.

## 4 Discussion

Across a 200‐km multidiscipline ultra‐endurance desert race in cool to warm environmental conditions, hypothermic (Tc min: 35.7°C) and hyperthermic (Tc max: 39.2°C) Tc's were observed. Despite an ∼12°C difference in day (28°C) versus. night ambient temperatures (16°C), Tc means were similar. GPS data were captured via the real‐time monitoring technology ecosystem throughout the race for 17 out of 18 participants, one smartphone battery ran out ∼6 h before race completion. The longest individual capture time was 57:32:04. Technological and connectivity issues/errors reduced the time physiological and biomechanical data were reliably displayed. Retrospective data analysis identified biometric variables were available in real‐time (and retrospectively) for ∼42% (∼38%–∼49%) of the participants on course time. One participant was medically evacuated which will be subsequently discussed in depth.

### 4.1 Thermoregulatory Responses

Increased Tsk and decreased Tc‐to‐Tsk gradient were observed during the daylight hours (see Figure 3C,D). This is unsurprising given solar radiation can add ∼100 W of additional heat load (Nielsen et al. 1988) inducing a greater thermal strain on an individual, resulting in increases in Tsk and reduced Tc‐to‐Tsk gradient (Otani et al. 2017). In the context of athlete health and performance, a lower Tc‐to‐Tsk gradient increases cardiovascular strain (Chou et al. 2018) and may be of greater detriment to performance than high Tc alone (Cuddy et al. 2014). This is attributable to a reduced capacity to dissipate heat from the core to the periphery and lower rates of convective heat loss. Thus, despite the similar mean and peak Tc between day and night during the SAMLA race, athletes were likely at a greater risk of EHI/EHS during daytime.

A mean Tc of 37.8°C is lower compared to previous ultra‐endurance events [161‐km Western States Endurance Run (WSER): 38.2°C (Valentino et al. 2016); Ironman triathlons (Hawaii/Western Australia): 38.1°C (Laursen et al. 2006) and 38.5°C (Olcina et al. 2019) and the 89‐km Comrades ultramarathon: 38.4°C (Byrne et al. 2022)] in similar environmental conditions [23°C–30°C (Byrne et al. 2022; Laursen et al. 2006; Olcina et al. 2019 and Valentino et al. 2016)]. Direct comparisons between previous ultra‐endurance research/events and the SAMLA race are made with caution due to the differences in exercise modality, distances covered and that SAMLA was a multiday event where many of the current sample slept or took extended rest at aid camps at the end of each stage. Tc at the start of each stage was similar (Table 2), suggesting Tc recovered (i.e., returned to baseline) between stages, a distinct difference between SAMLA and previous ultra‐endurance races (Byrne et al. 2022; Laursen et al. 2006; Olcina et al. 2019; Valentino et al. 2016). Furthermore, a lower intensity of exercise was exhibited by the participants during SAMLA. The mean race speed during SAMLA for all finishers was 5.4 km/h (3.5 km/h–11.3 km/h), mean HR was 115 b·min^−1^ (104 b·min^−1^–136 b·min^−1^) and mean %HRmax was 63% (53%–75%) compared to:

1. WSER: ∼6.0 km/h [no HR data (Valentino et al. 2016)]
2. Ironman triathlons: ∼22 km/h (Laursen et al. 2006; Olcina et al. 2019), 143 b·min^−1^, 83% HRmax (Laursen et al. 2006) and no HR data (Olcina et al. 2019)
3. Comrades ultramarathon: 8.6 km/h (7.4 km/h–10.6 km/h), 148 b·min^−1^ (133 b·min^−1^–158 b·min^−1^) and 79% (76%–84%) HRmax (Byrne et al. 2022)

Although a similar mean speed during the run stages mean Tc was lower during SAMLA than WSER (WSER: 38.2°C vs. SAMLA Run stages: 38°C and 37.9°C). The WSER includes elevation gains (5500m) and losses (7000m) that likely elicited greater physiological strain and increased metabolic heat production, compared to the flat desert terrain in Qatar [maximum elevation ∼200m (Ibrahim 2012)] and likely account for the difference in mean Tc. Despite the finishing times of the study participants being 12–40 h slower than that of the top 3 (mean speed: 11.1 km/h) and a low mean race speed (5.1 km/h), Tc exceeded 39°C during the run stages [mean speed Run 1: 5.0 km/h (3.9 km/h–7.5 km/h) and Run 2: 4.6 km/h (3.6 km/h–5.9 km/h)] where participants spent 72%–79% of the run stages walking. Without physiological data, event organisers/medical teams could view an event, such as SAMLA, as low risk from an EHI/EHS perspective given the warm but not extreme environmental conditions during SAMLA (compared to recent World Championship (Racinais et al. 2022) and Olympic (Tanaka et al. 2023) events) and low exercise intensity levels. Importantly, the current study demonstrates that significant thermal strain can still be experienced. Tc exceeded 39°C [a risk factor for heat‐related illnesses (Roberts et al. 2023)] and there were large increases in Tsk (Run 1 mean increase: 4.3°C and Run 2: 7.5°C) indicating EHI/EHS risk was still present (Roberts et al. 2023).

Ultra‐endurance research has focused on warm/hot events where EHI/EHS is a major risk (Singh et al. 2023). The SAMLA race has illustrated that ultra‐endurance events (particularly multiday events) may pose hypothermia concerns. The lowest Tc during SAMLA was observed during the swim, where cool/cold water temperatures are a significant risk factor for hypothermia for swimmers due to a ∼25‐fold increase in the thermal conductivity of water compared to air (M. Tipton and Bradford 2014). Furthermore, nighttime cooler temperatures [and no solar radiative heat load (Bouscaren et al. 2019)], and thus, lower Tsk and higher Tc‐to‐Tsk gradient (i.e., greater heat dissipation capacity) increases the risk of hypothermia although, exercise activity appears to have mitigated this during the SAMLA race alongside cool (16°C–18°C) but not cold ambient temperatures. Ultra‐endurance events where the contrast between day/night or sea‐level/altitude ambient temperatures are greater than those observed during SAMLA or ambient conditions change rapidly may pose a more severe hypothermia risk [e.g., previously outlined events in China (Hoffman 2021)].

### 4.2 Real‐Time Monitoring

A key aim of this study was to determine whether real‐time monitoring of athletes could be achieved across a 200‐km ultra‐endurance race where at least 57 h of data capture was required. Over 660 h of data, transmitted in one second intervals, were available in real‐time suggesting that the implementation of the technology was largely successful. Ultimately, the aim of this technology is to improve the safety of competitions by reducing the risk of participants suffering long‐term illness/injury and potential fatalities. During SAMLA 2023, 43% of race entrants DNF with a variety of injuries and voluntary withdrawals (specific data not available) with no serious incidents recorded. Over 12 years, 51 fatalities were recorded across mountain sports/races, including sudden cardiac arrest (43%), hypothermia (16%) and animal attacks [2% (Roi 2021)], up to 1.9 sudden cardiac deaths per 100,000 runners have been reported (Waite et al. 2016). Medical events, complications and injuries are common with 545 medical events recorded (8.27 incidents per 1000 starters) at the Two Oceans Marathon (21 and 56 km events) across four years, 37 of which were considered serious (Schwabe et al. 2014). In remote settings, minor injuries or health issues can quickly become serious and/or life‐threatening (Hoffman et al. 2020). It is simply not viable to cover the vast distances and remote locations within an ultra‐endurance event with the type of medical resource and staff implemented during marathon/race‐walk events in extreme heat. Data from short‐looped racecourses at the Doha 2019 World Athletics Championships (Racinais et al. 2022) and Tokyo 2020 Olympics (Tanaka et al. 2023) suggest that race medical teams are well‐prepared to deal with high incidences of EHI/EHS, 50 of the 100 EHI diagnoses at the Tokyo 2020 Olympics occurred during the marathon and race‐walking (Tanaka et al. 2023) and only five athletes required hospital treatment (Tanaka et al. 2023). Athletes suffering an EHI/EHS on short‐looped courses are quickly attended to and transported to the medical tent. Point‐to‐point racecourses, such as the Boston Marathon [51 cases of EHS 2015–19 (Breslow et al. 2021)], or other ultra‐endurance events (marathon des sables) means attending to medical events of any kind presents a greater challenge. GPS tracking of participants in these events is essential (Hoffman et al. 2020); however, without biometric parameters available to medical teams, the severity of a medical situation is unknown.

#### 4.2.1 Review of Medical Evacuation

One participant was hospitalised following an on‐course collapse. Participant follow‐up determined that they received treatment for exhaustion with other no medical issue(s) and were discharged within 24 h. Despite being instrumented with all sensors available in this study, his collapse would likely not have been predicted and/or identified earlier if medical staff had access to his data. At the time of collapse, his HR (120 b·min^−1^), Tc (38.1°C) and Tsk (33.4°C) data would not have raised alarm to medical staff. In the hour preceding collapse, maximum values from the available data would not have caused concern (HR: 151 b·min^−1^; Tc: 38.4°C and Tsk: 34.3°C). This case highlights the complexity of identifying and/or diagnosing those suffering an EHI based on a single physiological measure alone (Laitano et al. 2019; Roberts et al. 2023). Tolerance of heat stress is variable between individuals (Cheung et al. 2000; Westwood et al. 2021). Indeed, an EHI/S can occur in temperate environments without clinically recognised hyperthermic Tc (≥ 40°C), especially if Tc measurement is delayed (Epstein and Yanovich 2019). Through a multitude integrated physiological (i.e., high core and tissue temperature) and nonphysiological (e.g., medication/supplement use) factors (Westwood et al. 2021), including but not limited to physical fitness, previous EHI and/or illness [e.g., diarrhoea (Racinais et al. 2022)] and the accumulation of thermal load [e.g., multiple days (Westwood et al. 2021)]. In contrast to the medical evacuation in the current study, individuals can experience high Tc (≥ 40°C) and Tsk (≥ 36°C) without adverse effects (Henderson et al. 2023; Singh et al. 2023). Indeed, a Tc of 41.5°C was present in an elite road cyclist without any EHI symptomology (Racinais et al. 2019).

This real‐time monitoring ecosystem is constantly developing alongside wearable sensors that assess a variety of biometric data (including but not limited to: ECG, sweat rate/composition and interstitial glucose) which may enhance this system's ability to predict/alert medical teams to a medical emergency. Importantly, validity/reliability testing is required on all new technology before it should be adopted for in‐race monitoring. However, this incident demonstrates that medical incidents may not be predictable, even with real‐time biometric data, and medical teams must continue to operate on high alert to enable a rapid response to an emergency that may occur at any time.

#### 4.2.2 Real‐Time Monitoring Strengths and Limitations

Overall, real‐time monitoring of GPS and Tc/Tsk appears viable with good data acquisition and technological reliability. Data that suggest real‐time monitoring of ultra‐endurance athletes in‐competition can become a reality and provide race organizers/medical teams with data to make informed decisions on athlete health and safety in real‐time, optimally triage/manage their resources and thus, place athlete health and safety at the forefront of all sports‐related decision‐making (Adams 2024). For heart rate and gait, further work is required until they can be considered viable variables to be incorporated into a real‐time monitoring ecosystem. Realistically, before a system of this nature can be implemented widely, the limitations of this current real‐time ecosystem should be addressed:

- *Athlete burden:* Participants had to carry a smartphone, powerbank and gateway device (total: ∼650g) in their race backpack and powerbanks needed changing during the race to maintain battery life of the smartphone. Discomfort of the HR monitor mounted on a chest strap meant many participants chose not to use one and/or removed it from the chest during the race and a wearable alternative is required for ultra‐endurance events. Valid and reliable alternatives to chest belt are becoming available (e.g., arm‐bands and watches and/or rings using pulse oximetry) to measure heart rate; however, the quality of cardiac measurements is reportedly poor and cannot help in diagnosing potential heart issues (Etiwy et al. 2019).
- *Technological limitations:* Tc and Tsk measurements were limited to three sensors per participant. To be able to accurately assess Tsk, 2–6 sites are recommended to be within 1°C of actual Tsk (Taylor et al. 2014). Ideally, the telemetric Tc pill should be ingested 4–6 h prior to the start of a race; thus, an ultra‐endurance event of multiple days or even ≥ 6 h likely require the use of 2–3 pills to ensure continuous Tc monitoring and avoid data loss via excretion of the pill.
- *Data capture/logging:* During this study, server‐related issues and loss of data from excreted and/or broken sensors were a matter that impacted the amount of data that could be analysed retrospectively. Future developments should focus on ensuring retrospective data quality and the robustness of the ecosystem to retain all the data that is visualised in real‐time. Although this ecosystem is in its infancy and is not yet adopted by race medical teams, considerable data are required to demonstrate how/why this technology is important and how it can increase the safety of events in the future.

Thus, future developments should be focused on a single, small and lightweight device to transmit data from all sensors to the real‐time dashboard to alleviate the burden on athletes and increase/improve Tc/Tsk measurement capabilities. Postrace data availability should also be improved before a real‐time monitoring ecosystem can be deemed viable for use in competitions, particularly in elite sports events.

#### 4.2.3 Study Limitations

Future studies would benefit from addressing the limitations of this work. Collection of extraneous variables, such as hydration status, clothing worn and use of heat mitigation strategies (e.g., cooling), alongside accurate environmental data (e.g., temperature, humidity, solar radiation and wind speed), would better contextualise the thermoregulatory responses observed. The inclusion of female participants/athletes within ultra‐endurance competition research is imperative to determine how physiological responses differ from males. In hot conditions, females may suffer more rapid increases in Tc than males resulting from a lesser sweating capacity (Gagnon et al. 2013; Gagnon and Kenny 2011), higher body fat higher body fat (Cramer and Jay 2016), higher body surface area to mass ratio (Corbett, Wright and Tipton, 2023 and typically lower physical fitness (Kenney 1985) in comparison to males.

## 5 Conclusion

A 200‐km ultra‐endurance multidiscipline race elicited significant thermal strain (low Tc: 35.7°C and Tsk: 11.7°C; high Tc: 39.2°C and Tsk: 38.4°C) illustrating the unique thermoregulatory challenges ultra‐endurance sports pose that may exacerbate health risks despite only cool nighttime ambient temperatures and relatively low intensity exercise being exhibited by participants in warm but not extreme daytime temperatures. This study highlights how in‐race physiological responses could play an important role in protecting athlete health and performance, particularly in what may be deemed nonextreme race conditions, and in future allow race organisers to optimally manage their resources. The data were collected and visualised in one second intervals, in real‐time, with a total data capture of ∼660 h exceeding 57 h in individual cases. However, this technology is still in its infancy and continuous development and testing are needed to address the limitations observed in this study before it can be adopted on a large scale.

## Ethics Statement

Procedures were approved by the Aspire Zone Foundation Institutional Review Board (E202301049).

## Consent

Informed consent was obtained from all study participants prior to the start of the race.

## Conflicts of Interest

Y.P. is one of the founders of Human Telemetics Ltd.

## Data Availability

The data that support the findings of this study are available from the corresponding author upon reasonable request.
